# Early outcome prediction on 18F-fluorocholine PET/CT in metastatic castration-resistant prostate cancer patients treated with abiraterone

**DOI:** 10.18632/oncotarget.2558

**Published:** 2014-10-11

**Authors:** Ugo De Giorgi, Paola Caroli, Salvatore L. Burgio, Cecilia Menna, Vincenza Conteduca, Emanuela Bianchi, Francesca Fabbri, Elisa Carretta, Dino Amadori, Giovanni Paganelli, Federica Matteucci

**Affiliations:** ^1^ Department of Medical Oncology, Istituto Scientifico Romagnolo per lo Studio e la Cura dei Tumori (I.R.S.T.) IRCCS, Meldola, Italy; ^2^ Department of Nuclear Medicine, Istituto Scientifico Romagnolo per lo Studio e la Cura dei Tumori (I.R.S.T.) IRCCS, Meldola, Italy; ^3^ Biostatistics and Clinical Trials Unit, Istituto Scientifico Romagnolo per lo Studio e la Cura dei Tumori (I.R.S.T.) IRCCS, Meldola, Italy

**Keywords:** Abiraterone, Castration-resistant prostate cancer, 18F-fluorocholine positron emission tomography, PSA, Bone flare

## Abstract

Objective: We investigated the role of 18F-fluorocholine positron emission tomography/computed tomography (FCH-PET/CT) in the early evaluation of abiraterone and outcome prediction in patients with metastatic castration-resistant prostate cancer (CRPC).

Patient and methods: Forty-three patients with metastatic CRPC progressing after docetaxel received abiraterone 1,000 mg daily with prednisone 5 mg twice daily. Patients were evaluated monthly for serological PSA response and safety. FCH-PET/CT was done at baseline and after 3 to 6 weeks. Univariate and multivariate Cox regression models addressed potential predictors of progression-free survival (PFS) and overall survival (OS).

Results: Declines in PSA level of ≥50% were seen in 21 of 43 (49%) patients. Forty-two patients were evaluable for FCH-PET/CT response. FCH-PET/CT bone flare was observed in 4 of 42 (10%) evaluable patients. In univariate analysis, PSA decline and FCH-PET/CT response predicted PFS, while PSA decline and FCH-PET/CT (progression vs non progression) predicted OS. In multivariate analysis, only FCH-PET/CT (progression vs nonprogression) remained significant for PFS and OS (*p* = 0.022 and *p* = 0.027, respectively).

Conclusion: Early FCH-PET/CT can predict clinical outcome in CRPC beyond PSA response. These data support further studies on FCH-PET/CT for abiraterone monitoring and outcome prediction in patients with CRPC.

## INTRODUCTION

A well-recognized barrier to therapy management in castration-resistant prostate cancer (CRPC) is the lack of reliable surrogate markers of response to treatment coupled with a potentially exaggerated reliance on changes in serum prostate-specific antigen (PSA) as an indicator of treatment efficacy. This is particularly true with agents that have the potential for PSA modulation such as abiraterone, a selective inhibitor of androgen biosynthesis that potently and irreversibly blocks cytochrome P-450c17 (CYP17), which improved survival in metastatic CRPC [1–3]. Consequently, changes in radiological images have been advocated as important markers of disease response and progression to abiraterone [4, 5].

Altered choline metabolism is a characteristic of prostate cancer, and 18F-fluorocholine Positron emission tomography (PET) - computed tomography (CT) (FCH-PET/CT) is one of the most promising tools for prostate cancer imaging [6]. FCH-PET/CT has been shown to have a higher sensitivity and specificity than standard 99m Tc-MDP bone scans and 18F- fluorodeoxyglucose (FDG)-PET/CT for the detection of bone metastases from CRPC [7–9]. On the basis of these characteristics, this metabolic imaging might be considered for further evaluation for guiding prostate cancer management [10–12].

We investigated the role of FCH-PET/CT in the early treatment evaluation and outcome prediction in patients with CRPC treated with abiraterone.

## RESULTS

### Patient characteristics

Between October 2011 and August 2012, 43 CRPC patients were considered for this study with PSA measurement and FCH-PET/CT imaging studies performed at baseline before starting abiraterone. At baseline, all patients had evidence of metastatic CRPC at FCH-PET/CT, 39 had bone metastases. Characteristics of the 43 patients included are summarized in Table 1. Therapy-related toxicity with treatment interruption were pulmonary thromboembolism (n = 1), grade 3 hypertransaminasis (n = 1), cardiac heart failure (n = 1), while no abiraterone-related therapy interruption were infective diarrhea (n = 1) and renal failure (n = 1). Other grade 1–2 reported side effects were: asthenia (n = 23, 53%), anemia (n = 19, 44%), diarrhea (n = 7, 16%), nausea (n = 5, 12%), fluid retention (n = 2, 4%), hypertension n=1 (2%).

**Table 1:** Demographic and clinical characteristics of patients with castration-resistant prostate cancer at baseline (n = 43)

Characteristic	N (%)
**Median age (range), years**	73 (57–87)
**ECOG Performance status**	
0–1	42 (98%)
2	1 (2%)
**Gleason score**	
6–7	15 (35%)
8–9	26 (60%)
Unknown	2 (5%)
**Sites of disease**	
Bone	39 (91%)
Node	24 (56%)
Lung	2 (5%)
Liver	1 (2%)
**No. of previous chemotherapeutic regimens**	
One	27 (63%)
Two or more	16 (37%)
**Baseline PSA**	
Median (range), ng/mL	23.3 (1.5–1083)

### Relationship between PSA decline and therapy response at FCH-PET/CT

Declines in PSA level of 50% or more were observed in 21 of 43 (49%) patients. The CT response at 3 months of follow-up was assessed in 42 patients, while one case with renal dysfunction did not perform it. 3-month CT results were: complete response (n = 1, 2%), partial response (n = 1, 2%), stable disease (n = 28, 67%), PD (n = 12, 29%). Forty-two patients were evaluable for FCH-PET/CT response after a median time between baseline and follow-up FCH-PET/CT scanning of 40 days (range, 19 to 69 days). One of 43 patients did not perform the follow-up FCH-PET/CT due to rapid PD with clinical deterioration and decline in performance status.

**Figure 1:**
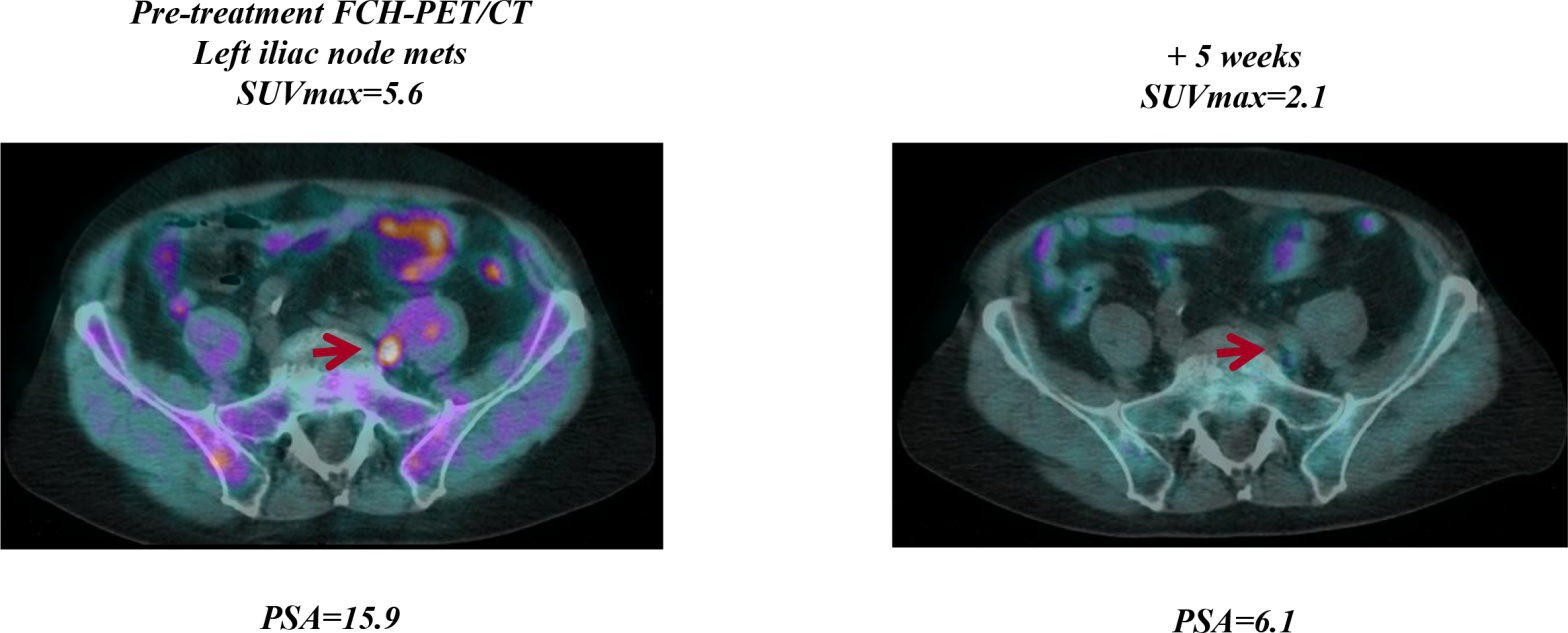
Early FCH-PET/CT complete response on abiraterone treatment Baseline FCH-PET/CT shows a single left iliac nodal lesion, 5 week FCH-PET/CT resolution correlated with PSA decline and then with undetectable PSA levels (PSA < 0.03 ng/mL) after 6 months of abiraterone therapy.

**Figure 2:**
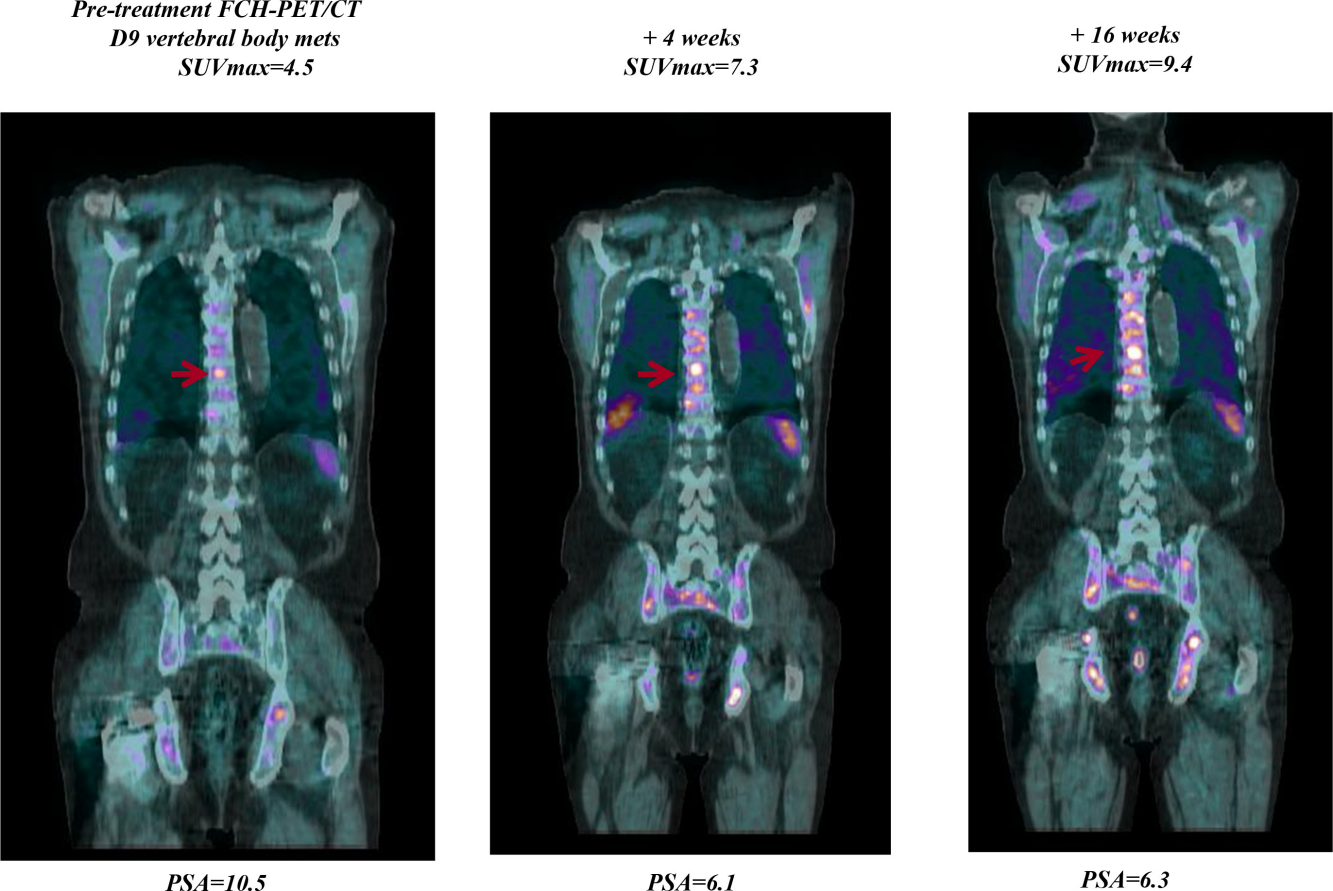
Early FCH-PET-CT progression on abiraterone treatment Baseline FCH-PET/CT shows multiple areas of increased radiotracer uptake indicative of extensive bone metastases. Treatment with abiraterone resulted in bone progression of FCH-PET/CT scans at week 4 confirmed at week 16.

**Figure 3:**
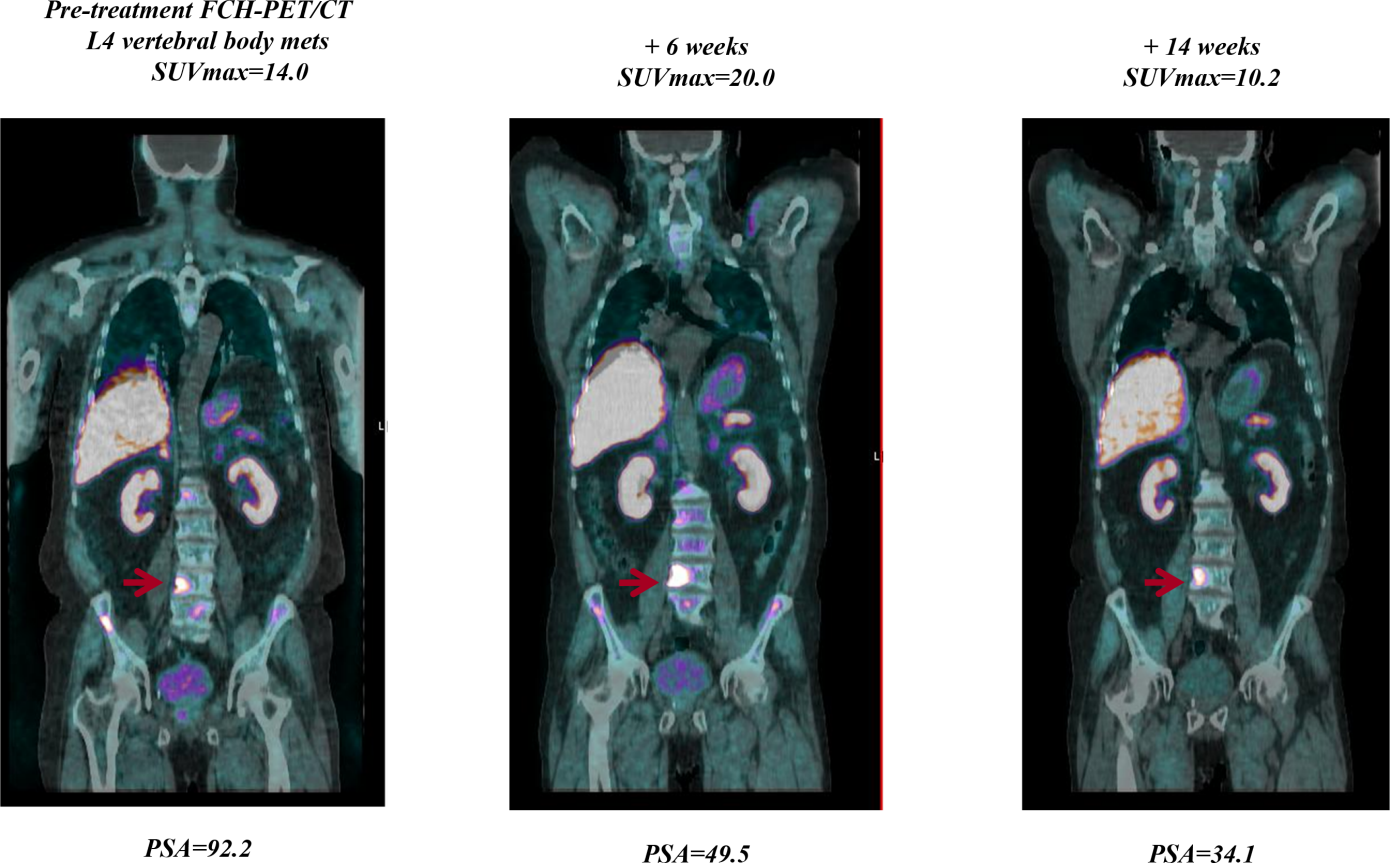
Example of a patient with a rapidly declining PSA level but a week 6 FCH-PET/CT being read as progression in bone lesions By week 14, this progression improved, indicating that the progression seen at week 6 was due to bone flare.

FCH-PET/CT scanning showed 3 CR, 11 PR, 6 SD, and 22 PD, but in 6 of 12 pts with initial PD at FCH-PET/CT a bone flare phenomenon was suspected due to a stable disease at 3-month CT scan associated with a progressive decline of PSA. In one case suspected for bone flare, the PET scan was not repeated due to severe intestinal infection with treatment interruption and following clinical deterioration. In another patient who had a 3-month PSA decline of 60% with SD at 3-month CT scan, the follow-up FCH-PET/CT scanning after 11 weeks confirmed PD with rapidly increasing PSA levels. Thus, overall, bone scan flare, as defined by the combination of PSA decline, initial flare, and subsequent improvement or stability in the following FCH-PET/CT scanning was observed in 4 of 42 (10%) evaluable patients and 4 of 24 (17%) responsive cases. The clinical characteristics of these 4 cases were not significantly different from the study population as a whole, median age was 71 years (range, 57 to 78), median PSA level at baseline was 15.5 ng/mL (range, 1.5–92.2), the maximal decline of PSA was 91%, 73%, 64% and 37%, respectively. The latter case who had a very low baseline PSA level of 1.5 ng/mL and PSA decline to 0.9 ng/mL (37%, lower than the threshold of 50%) was also considered to be of interest and is reported as bone flare. A PSA flare, defined as an initial PSA increment followed by a decline below the PSA baseline level, was reported in one case of bone flare, but also in another patient without bone flare. Of 4 patients with FCH-PET/CT bone flare, 3 had PD after 9, 11 and 13 months, respectively, while one is continuously progression free after 22+ months. A CR was achieved at follow-up FCH-PET/CT in 3 of the 26 (12%) evaluable patients, and was associated with undetectable PSA levels (< 0.03 ng/mL) in 1 case after 5 months on abiraterone. Of 3 patients with nodal disease only, one obtained a CR at first FCH-PET/CT and 2 a PR with a mean SUVmax reduction of 55% and 70%, respectively. Figure 1 to 3 show examples of early FCH-PET/CT effects of abiraterone, including an example of bone flare.

Changes in PSA levels were concordant with the FCH PET/CT response/non-response in 30 of 42 patients (71%) (Table 2), and with the FCH-PET/CT progression/non-progression in 34 of 42 (81%) (Table 3).

**Table 2:** Comparison of PSA decline and FCH PET/CT in responding vs nonresponding patients

Response at Follow-up FCH PET/CT Imaging Study^a^	PSA Response		Total
	PSA <50%	PSA >50%	
Response	12^b^ (75%)	4^c^ (25%)	16 (38%)
Nonresponse	8^d^ (31%)	18 (69%)	26 (62%)
Total	20 (48%)	22 (52%)	42 (100%)

a Response was defined as complete response or partial remission; nonresponse was defined as stable disease or progressive disease.

b Including 2 patients with bone flare at FCH PET/CT with SUV decline at second-follow-up PET >25%, and 1 patient with partial remission at PET but liver progression at 3-month CT only.

c Including 1 patient with bone flare at at FCH PET/CT with PSA decline = 37.2% after 6 months of available follow-up.

d Including 1 patient with bone flare at at FCH PET/CT with PSA decline = 48.8%, in1 patient suspected for bone flare, but PET scan was not repeated due to severe intestinal infection with treatment interruption and clinical deterioration.

**Table:** 

		Lower	Upper
Measurement	Estimate	95% CI	95% CI
Positive % Agreement	60%	36%	81%
Negative % Agreement	82%	60%	95%
Positive Predictive Value	75%	48%	93%
Negative Predictive Value	69%	48%	87%
Overall Agreement	71%	55%	84%
Odds Ratio	6.75	1.66	27.51

**Table 3:** Comparison of PSA decline and FCH PET/CT in non-progressing vs progressing patients

Response at Follow-up FCH PET/CT Imaging Study^a^	PSA Response		Total
	PSA <50%	PSA >50%	
NonProgression	18^b^ (75%)	6^c^ (25%)	24 (57%)
Progression	2^d^ (11%)	16 (89%)	18 (43%)
Total	20 (48%)	22 (52%)	42 (100%)

a Nonprogression was defined as complete response, partial remission or stable disease; progression was defined as progressive disease.

b Including 3 patients with bone flare at FCH PET/CT, and 1 patient with partial remission at PET but liver progression at 3-month CT only.

c Including 1 patient with bone flare at at FCH PET/CT with PSA decline = 37.2% after 6 months of available follow-up, and one patient with PSA decline = 48.8%.

d 1 patient suspected for bone flare, but PET scan was not repeated due to severe intestinal infection with treatment interruption and clinical deterioration.

**Table:** 

		Lower	Upper
Measurement	Estimate	95% CI	95% CI
Positive % Agreement	90%	68%	99%
Negative % Agreement	73%	50%	89%
Positive Predictive Value	75%	53%	90%
Negative Predictive Value	89%	65%	99%
Overall Agreement	81%	66%	91%
Odds Ratio	24	4.23	136.22

**Figure 4:**
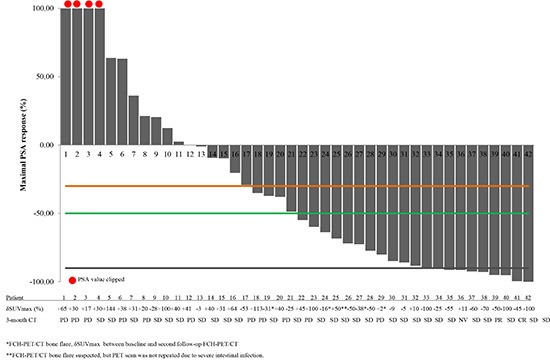
Changes in PSA levels and correlation with either follow-up FCH-PET/CT and CT scan in 42 castration-resistant prostate cancer patients treated with abiraterone with assessable follow-up FCH-PET/CT Waterfall plots of maximal PSA change. Reference lines: black, −90%; orange, −50%; green, −30%. Mean change in maximum standardized uptake value (δSUVmax). The table shows the mδSUVmax and 3-months CT response of corresponding patients. Patients with FCH-PET/CT bone flare are marked with an asterisk (*); in these cases δSUVmax is calculated between baseline and second follow-up FCH-PET/CT. A case of suspected FCH-PET/CT bone flare is marked with two asterisks (**); in this case PET scan was not repeated due to severe intestinal infection with clinical deterioration. CT scan response (RECIST criteria).

Figure 4 describes waterfall plots of maximal PSA change and correlation with either follow-up FCH-PET/CT and CT scan in 42 evaluable patients.

### Ability to predict survival

In all 43 patients, the median PFS time was 7.0 months (95% CI: 5.4 – 11.0), while the median OS time was 18.7 months (95% CI: 10.8 – …). At the time of analysis, after a median follow-up time of 22 months, 13 patients (30%) were considered progression free, whereas 17 (40%) patients had died. Patients with nonprogression at FCH-PET/CT and without bone flare had a median PFS of 12.8 months, while those with bone flare had a PFS of 11.0 months (*p* = 0.769). Patients with progression at FCH-PET/CT had a median PFS of 3.4 months, statistically different from PFS of those with nonprogression at FCH-PET/CT without or with bone flare (*p* = 0.0001 and *p* = 0.0033, respectively).

Univariate and multivariate analyses were performed to assess the association between factors of interest and PFS and OS. In univariate analysis, PSA decline, FCH-PET/CT (SD/PD vs PR/CR) and FCH-PET/CT (progression vs non progression) predicted PFS, while PSA decline and FCH-PET/CT (progression vs non progression) predicted OS (Table 4). In multivariate analysis, only FCH-PET/CT (progression vs nonprogression) remained significant for PFS and OS (*p* = 0.022 and *p* = 0.027, respectively) (Table 4).

**Table 4:** Univariate and multivariate Cox regression analyses for PFS and OS in patients with castration-resistant prostate cancer treated with abiraterone

Univariate Analysis	PFS Risk from Baseline			OS Risk from Baseline		
Parameters	HR	95% CI	*P*	HR	95% CI	*P*
Gleason score (8–9 vs 6–7)	1.0	0.5 – 2.2	0.985	1.8	0.6 – 5.3	0.273
No. previous chemotherapeutic regimens (>2 vs 1)	1.8	0.8 – 3.8	0.126	2.4	0.9 – 6.7	0.088
PSA decline (<50% vs >50%)	3.6	1.6 – 7.9	0.002	3.3	1.2 – 9.2	0.022
FCH-PET/CT (SD/PD vs CR/PR)	3.1	1.3 – 7.4	0.009	2.8	0.9 – 8.8	0.078
FCH-PET/CT (PD vs CR/PR/SD)	6.8	2.7 – 17.5	<0.0001	5.3	1.8 – 15.1	0.002

Multivariate Analysis	PFS Analysis			OS Analysis		
Parameters	HR	95% CI	*P*	HR	95% CI	*P*
PSA decline (<50% vs >50%)	2.0	0.8 – 8.1	0.134	1.4	0.4 – 5.1	0.568
FCH-PET/CT (SD/PD vs CR/PR)	1.3	0.4 – 4.2	0.623			
FCH-PET/CT (PD vs CR/PR/SD)	4.1	1.2 – 14.0	0.022	4.3	1.2 – 15.4	0.027

PFS = progression-free survival; OS = overall survival; HR = hazard ratio; CI = confidence interval; SD = stable disease; PD = progressive disease; CR = complete response; PR = partial remission.

## DISCUSSION

To the best of our knowledge, this is the first study that used FCH-PET/CT for outcome prediction and tumor monitoring in CRPC. The correlation between the prognostic value of PSA and FCH-PET/CT response to therapy suggests that PSA levels are linked to a tumor metabolic activity. Furthermore, in multivariate analysis FCH-PET/CT (progression vs nonprogression) was superior to PSA with respect to PFS and OS prediction, supporting the hypothesis of an essential role of FCH-PET/CT to improve therapy management.

PSA decline ≥50% concurred with the FCH-PET/CT response/non-response in 71% of patients (Table 2), and with the FCH-PET/CT progression/non-progression in 22 out of 26 81% cases (Table 3). Moreover, in univariate analysis, FCH-PET/CT (SD/PD vs PR/CR) and FCH-PET/CT (progression vs non progression) predicted PFS, whereas in multivariate analysis only FCH-PET/CT (progression vs nonprogression) remained significant for PFS and OS (*p* = 0.022 and *p* = 0.027, respectively) (Table 4). However, a main limitation of this study is represented by the criteria used for FCH-PET/CT classification of response, based on fairly arbitrary thresholds for change in SUV (as specified in EORTC guidelines). In this regard the differentiation between responders and non-responders results much less reliable than the FCH-PET/CT progression/non-progression evaluation.

PET/CT progression/non-progression proved capable of predicting outcome in patients with other tumors, including bone metastases [13, 14]. As abiraterone in CRPC is given continuously until progression, it is essential to predict and monitor progression rather than the response to treatment. PET/CT criteria for monitoring tumor progression are simpler and more widely accepted than those for monitoring tumor response. The relationship of PSA kinetics to radiological PFS has been recently evaluated in an exploratory analysis of patients treated with abiraterone in a randomized phase III study [15]. Radiological PFS was positively associated with the magnitude of PSA decline, and the effect remained after correcting for covariates. However, in this trial the PSA level was monitored every 2–3 months, whereas the kinetics of serum PSA analyzed monthly during the first few months of abiraterone is associated with PSA flare in nearly 10% of these patients, which could impair the early evaluation of PSA response [16], The impact of FCH-PET/CT could be even more relevant for CRPC with no reliable serum biomarker, as is the case of bulky metastatic disease with low serum PSA levels. However, in a few of these cases, elevated circulating Chromogranin A levels have been reported as possible expression of a CRPC with neuroendocrine differentiation in which the role of FCH-PET/CT is not known [17, 18]. Thus, the integrated use of PSA and FCH-PET/CT could potentially lead to a significant improvement in outcome prediction and in the therapeutic monitoring of CRPC patients.

Clinical studies have demonstrated that PET can predict response to chemotherapy and targeted therapy in cases of several tumors [14, 19–21]. The early identification of therapeutic response supports the clinician in the decision making. Moreover, new functional imaging techniques such as whole-body diffusion weighted magnetic resonance imaging (MRI) can improve the evaluation of therapy response in patients with metastatic bone disease with a potential significant impact in the therapy assessments of patients with prostate cancer because of the high prevalence of bone-only disease [23, 24].

Controversy still exists on the applicability of PET/CT and other new imaging tools in clinical practice, mainly because of its expense. However apart from additional costs related to PET/CT examinations, potential savings associated with PET/CT as a result of avoiding additional imaging examinations and by helping clinicians make the optimum treatment decisions avoiding that multiple doses of high-cost antineoplastic agents are administered, even if they are ineffective. Thus, the potential implementation in clinical practice of FCH-PET/CT as well as predictor of clinical outcome needs further evaluation and validation with larger patient population including cost-benefit analyses. Additionally alternative imagines such as whole-body diffusion weighted magnetic MRI should be also evaluated in the same contest and for the same purpose.

An important observation that emerges from our study is the evidence of the mismatch highlighted in 4 patients between the reduction of the value of serum PSA and the detection of an increased uptake of bone lesions shown in the PET/CT after 1–2 months. This mismatch was evaluated as related to the phenomena of "bone flare", already reported in patients after treatment with abiraterone in chemotherapy-naive CRPC who had undergone bone scintigraphy, but that stands out for the first time also with FCH-PET/CT [25]. FCH-PET/CT bone flare predicted a favorable long-term outcome similar to that of FCH-PET/CT non progressive patients. The PCWG2 consensus criteria considered bone flare and included the recommendation to perform a first follow-up bone scan 12 or more weeks after the start of treatment, and furthermore defines progression in bone when a minimum of 2 new lesions are observed and confirmed on a second scan done 6 or more weeks later [5].

The metabolic tumor changes as detected with FCH-PET/CT make possible new ways of managing and investigating CRPC. However, the early FCH-PET/CT bone flare phenomenon should be considered as a limitation ofits utility as an early indicator marker, or thefirst follow-up FCH-PET/CT should be carried out 3 months after the start of therapy. Moreover, FCH uptake may be influenced by several factors, including the number of viable cells per unit volume, the tumor vascular supply, the sites of metastases and the neuroendocrine differentiation [17, 18, 26, 27]. Recently, new PET tracers such as radiolabeled dihydrotestosterone and radiolabeled antibody to prostate-specific membrane antigen (PSMA) have shown great potential for assessing patients' early biologic response to hormonal therapies in prostate cancer [28–30].

In conclusion, in this pilot study early FCH-PET/CT was able to predict clinical outcome in CRPC beyond PSA response. Further larger studies are needed to better define the most sensitive and cost-effective modality for assessment of therapy efficacy in CRPC.

## PATIENTS AND METHODS

### Patients

Forty-three consecutive patients with advanced prostate cancer, without neuroendocrine differentiation, after radically prostatectomy and in progression on androgen deprivation, were enrolled. Patients previously received docetaxel and not more than two chemotherapy regimens for metastatic CRPC. Prior ketoconazole therapy was not permitted. Additional eligibility criteria included adequate performance status, cardiac, renal, hepatic and bone marrow function, serum potassium level 3.5 mmol/L or more, and ongoing androgen deprivation with serum testosterone <50 ng/dL. The protocol was approved by the Institutional Review Board. Written informed consent was obtained from all patients.

### Treatment and evaluation

Treatment consisted of abiraterone acetate 1,000 mg daily with prednisone 5 mg twice daily in 28-day cycles. Treatment was given continuously until there was evidence of progressive disease (PD) or unacceptable toxicity. PD was defined according to Prostate Cancer Working Group 2 (PCWG2) criteria [5]. Baseline examinations included PSA, CT and FCH-PET/CT scanning. Patients were evaluated monthly for serological PSA response and safety. FCH-PET/CT was repeated after 3 to 6 weeks. A CT scan was done after 3 months of abiraterone therapy.

Adverse events were graded using the National Cancer Institute Common Terminology Criteria for Adverse Events (CTCAE), version 4.

### PET/CT Imaging Protocol

FCH-PET/CT scans were performed on an integrated PET/CT system (Discovery Discovery LS camera, General Electric Medical Systems, Waukesha, WI) in 2D acquisition mode for 3 minutes per bed position. PET scanning was performed 45 minutes after intravenous injection of 18F-Methilcholine (3,7 MBq/Kg of body weight, AAA-Advanced Application Accellerator, Meldola, Italy). The field of view included the skull to mid- femurs. Low dose CT (120 kV, 80 mA) without contrast agents was performed for attenuation correction and as an anatomical map. The emission data were corrected for scatter, random coincidence events, and system dead time using the provided software.

### Image Reading and Interpretation

PET/CT scans were evaluated by two nuclear medicine physicians with consolidate experience. In case of disagreement between the readers, studies were reexamined and a consensus was reached. Criteria to define PET/CT positivity included the presence of focal areas of increased tracer uptake with or without any underlying lesion identified using CT. Semiquantitative criteria based on the maximum standardized uptake value (SUVmax) and the target to background ratio were used to aid the visual analysis [31]. Images were read sequentially by using a Xeleris III workstation (General Electric Medical Systems). PET, CT, and PET/CT fused images were used to correctly evaluate the scans in various cuts (axial, sagittal, and coronal)

### Measurement of treatment effect by FCH-PET/CT

Baseline FCH-PET/CT was performed in all patients and a minimum of 2 target lesions were defined as a references. Patients scanned 3–6 weeks after abiraterone therapy first cycle, were considered responders according to guidelines for PET on the basis of a metabolic activity decrease more than 25% of target lesions depicted at baseline images, coupled with or without evidence of change/decrease in size [32, 33]. A complete disappearance of FCH uptake was considered indicative of a metabolic complete response (CR), whereas less than 100% but more than 25% of FCH uptake was considered a metabolic partial remission (PR). Non-responders were the patients with similar or higher FCH uptake in the target lesions, and/or with a substantial increase of the target lesions size (ie, 20% increase in sums of longest diameters) [33]. Among nonresponders, increased FCH uptake in a target lesion that was also enlarged on CT imaging (ie, 20% increase in the longest diameter) and/or a new metabolically active lesion were considered indicative of progressive disease (PD), whereas a substantially unchanged FCH uptake without substantial increase in size was considered stable disease (SD).

In the statistical analysis of survival, we considered FCH-PET/CT responders (CR/PR) versus nonresponders (SD/PD), and progressing (PD) versus non-progressing disease (CR/PR/SD).

### FCH-PET/CT bone flare

FCH-PET/CT bone flare was defined as the combination of increased >25% uptake of known lesions at early FCH-PET/CT after 3–6 weeks of therapy, which occurred in the context of a ≥50% decline in PSA and no evidence of PD at 3-month CT scan, with FCH-PET/CT repeated 2–3 months later showing improvement or stability. An initial PET scan flare followed by continued declines in PSA levels below the threshold of 50% and stable uptake at the third FCH PET-TC was also considered to be of clinical interest.

### Statistical analysis

PFS was defined as the time elapsed between the date of the start of abiraterone and the date of PD or death or the date of the last follow-up visit [5]. OS was defined as the elapsed time between the date of the start of abiraterone and the date of either death or the last follow-up. Kaplan-Meier survival plots were generated on the basis of FCH-PET/CT response, and the curves were compared using log-rank test. Cox proportional hazards regression was used to determine univariate and multivariate hazard ratios for selected potential predictors of PFS and OS. A *p* value of < 0.05 was considered statistically significant.

## References

[1] Attard G, Reid AH, A'Hern R, Parker C, Oommen NB, Folkerd E, Messiou C, Molife LR, Maier G, Thompson E, Olmos D, Sinha R, Lee G, Dowsett M, Kaye SB (2009). Selective inhibition of CYP17 with abiraterone acetate is highly active in the treatment of castration-resistant prostate cancer. J Clin Oncol.

[2] Ryan CJ, Smith MR, de Bono JS, Molina A, Logothetis CJ, de Souza P, Fizazi K, Mainwaring P, Piulats JM, Ng S, Carles J, Mulders PF, Basch E (2013). Abiraterone in metastatic prostate cancer without previous chemotherapy. N Engl J Med.

[3] Fizazi K, Scher HI, Molina A, Logothetis CJ, Chi KN, Jones RJ, Staffurth JN, North S, Vogelzang NJ, Saad F, Mainwaring P, Harland S, Goodman OB (2012). Abiraterone acetate for treatment of metastatic castration-resistant prostate cancer: final overall survival analysis of the COU-AA-301 randomised, double-blind, placebo-controlled phase 3 study. Lancet Oncol.

[4] de Bono JS, Logothetis CJ, Molina A, Fizazi K, North S, Chu L, Chi KN, Jones RJ, Goodman OB, Saad F, Staffurth JN, Mainwaring P, Harland S (2011). Abiraterone and increased survival in metastatic prostate cancer. N Engl J Med.

[5] Scher HI, Halabi S, Tannock I, Morris M, Sternberg CN, Carducci MA, Eisenberger MA, Higano C, Bubley GJ, Dreicer R, Petrylak D, Kantoff P, Basch E (2008). Design and end points of clinical trials for patients with progressive prostate cancer and castrate levels of testosterone: recommendations of the Prostate Cancer Clinical Trials Working Group. J Clin Oncol.

[6] DeGrado TR, Baldwin SW, Wang S, Orr MD, Liao RP, Friedman HS, Reiman R, Price DT, Coleman RE (2001). Synthesis and evaluation of 18F-labeled choline analogs as oncologic PET tracers. J Nucl Med.

[7] Beheshti M, Vali R, Waldenberger P, Fitz F, Nader M, Loidl W, Broinger G, Stoiber F, Foglman I, Langsteger W (2008). Detection of bone metastases in patients with prostate cancer by 18F fluorocholine and 18F fluoride PET-CT: a comparative study. Eur J Nucl Med Mol Imaging.

[8] Umbehr MH, Müntener M, Hany T, Sulser T, Bachmann LM (2013). The role of 11C-choline and 18F-fluorocholine positron emission tomography (PET) and PET/CT in prostate cancer: a systematic review and meta-analysis. Eur Urol.

[9] Price DT, Coleman RE, Liao RP, Robertson CN, Polascik TJ, DeGrado TR (2002). Comparison of [18F]fluorocholine and [18F]fluorodeoxyglucose for positron emission tomography of androgen dependent and androgen independent prostate cancer. J Urol.

[10] Bauman G, Belhocine T, Kovacs M, Ward A, Beheshti M, Rachinsky I (2012). 18F-fluorocholine for prostate cancer imaging: a systematic review of the literature. Prostate Cancer Prostatic Dis.

[11] Langsteger W, Balogova S, Huchet V, Beheshti M, Paycha F, Egrot C, Janetschek G, Loidl W, Nataf V, Kerrou K, Pascal O, Cussenot O, Talbot JN (2011). Fluorocholine (18F) and sodium fluoride (18F) PET/CT in the detection of prostate cancer: prospective comparison of diagnostic performance determined by masked reading. Q J Nucl Med Mol Imaging.

[12] Kwee SA, Coel MN, Ly BH, Lim J (2009). (18)F-Choline PET/CT imaging of RECIST measurable lesions in hormone refractory prostate cancer. Ann Nucl Med.

[13] De Giorgi U, Valero V, Rohren E, Dawood S, Ueno NT, Miller MC, Doyle GV, Jackson S, Andreopoulou E, Handy BC, Reuben JM, Fritsche HA, Macapinlac HA (2009). Circulating tumor cells and [18F]fluorodeoxyglucose positron emission tomography/computed tomography for outcome prediction in metastatic breast cancer. J Clin Oncol.

[14] De Giorgi U, Mego M, Rohren EM, Liu P, Hndy BC, Reuben JM, Macapinlac HA, Hortobagyi GN, Cristofanilli M, Ueno NT (2010). 18F-FDG PET/CT findings and circulating tumor cell counts in the monitoring of systemic therapies for bone metastases from breast cancer. J Nucl Med.

[15] Ryan CJ, Londhe A, Molina A, Smith MR, De Bono JS, Mulders P, Rathkopf DE, Saad F, Logothetis C, Fizazi K, Scher HI, Small EJ, Matheny S (2013). Relationship of baseline PSA and degree of PSA decline to radiographic progression-free survival (rPFS) in patients with chemotherapy-naive metastatic castration-resistant prostate cancer (mCRPC): Results from COU-AA-302 [abstr 5010]. J Clin Oncol.

[16] Burgio SL, Conteduca V, Rudnas B, Carrozza F, Campadelli E, Bianchi E, Fabbri P, Montanari M, Carretta E, Menna C, De Giorgi U (2014). PSA flare with abiraterone in patients with metastatic castration-resistant prostate cancer. Clin Genitourin Cancer.

[17] Bollito E, Berruti A, Bellina M, Mosca A, Leonardo E, Tarabuzzi R, Cappia S, Ari MM, Tampellini M, Fontana D, Gubetta L, Angeli A, Dogliotti L (2001). Relationship between neuroendocrine features and prognostic parameters in human prostate adenocarcinoma. Ann Oncol.

[18] Burgio SL, Conteduca V, Menna C, Carretta E, Rossi L, Bianchi E, Kopf B, Fabbri F, Amadori D, De Giorgi U (2014). Chromogranin A predicts outcome in prostate cancer patients treated with abiraterone. Endocr Relat Cancer.

[19] Juweid ME, Cheson BD (2006). Positron-emission tomography and assessment of cancer therapy. N Engl J Med.

[20] Gayed I, Vu T, Iyer R, Johnson M, Macapinlac H, Swanston N, Podoloff D (2004). The role of 18F-FDG PET in staging and early prediction of response to therapy of recurrent gastrointestinal stromal tumors. J Nucl Med.

[21] Ell PJ (2006). The contribution of PET/CT to improved patient management. Br J Radiol.

[22] Herrmann K, Buck AK, Schuster T, Abbrederis K, Blümel C, Santi I, Rudelius M, Wester HJ, Peschel C, Schwaiger M, Dechow T, Keller U (2014). Week one FLT-PET response predicts complete remission to R-CHOP and survival in DLBCL. Oncotarget.

[23] Reischauer C, Froehlich JM, Koh DM, Graf N, Padevit C, John H, Binkert CA, Boesiger P, Gutzeit A (2010). Bone metastases from prostate cancer: assessing treatment response by using diffusion-weighted imaging and functional diffusion maps—initial observations. Radiology.

[24] Padhani AR, Makris A, Gall P, Collins DJ, Tunariu N, de Bono JS (2014). Therapy monitoring of skeletal metastases with whole-body diffusion MRI. J Magn Reson Imaging.

[25] Ryan CJ, Shah S, Efstathiou E, Smith MR, Taplin ME, Bubley GJ, Logothetis CJ, Kheoh T, Kilian C, Haqq CM, Molina A, Small EJ (2011). Phase II study of abiraterone acetate in chemotherapy-naive metastatic castration-resistant prostate cancer displaying bone flare discordant with serologic response. Clin Cancer Res.

[26] Mertens K, Slaets D, Lambert B, Acou M, De Vos F, Goethals I (2010). PET with 18F-labelled choline-based tracers for tumour imaging: a review of the literature. Eur J Nucl Med Mol Imaging.

[27] Conteduca V, Aieta M, Costantini M, Amadori D, De Giorgi U (2014). Current and emerging strategies on neuroendocrine prostate cancer. Crit Rev Oncol Hematol.

[28] Larson SM, Morris M, Gunther I, Beattie B, Humm JL, Akhurst TA, Finn RD, Erdi Y, Pentlow K, Dyke J, Squire O, Bornmann W, McCarthy T (2004). Tumor localization of 16beta-18F-fluoro-5alpha-dihydrotestosterone versus 18F-FDG in patients with progressive metastatic prostate cancer. J Nucl Med.

[29] Chen Y, Pullambhatla M, Foss CA, Byun Y, Nimmagadda S, Senthamizhchelvan S, Sgouros G, Mease RC, Pomper MG (2011). 2-(3-{1-carboxy-5-[(6-[18F]fluoro-pyridine-3-carbonyl)-amino]-pentyl}-ureido)-pentanedioicacid[18F]DCFPyL, a PSMA-based PET imaging agent for prostate cancer. Clin Cancer Res.

[30] Evans MJ, Smith-Jones PM, Wongvipat J, Navarro V, Kim S, Bander NH, Larson SM, Sawyers CL (2011). Noninvasive measurement of androgen receptor signaling with a positron-emitting radiopharmaceutical that targets prostate-specific membrane antigen. Proc Natl Acad Sci U S A.

[31] Mawlawi O, Podoloff DA, Kohlmyer S, Williams JJ, Stearns CW, Culp RF, Macapinlac H (2004). Performance characteristics of a newly developed PET/CT scanner using NEMA standards in 2D and 3D modes. J Nucl Med.

[32] Young H, Baum R, Cremerius U, Herholz K, Hoekstra O, Lammertsma AA, Pruim J, Price P (1999). Measurement of clinical and subclinical tumour response using [18F]-fluorodeoxyglucose and positron emission tomography: Review and 1999 EORTC recommendations—European Organization for Research and Treatment of Cancer (EORTC) PET Study Group. Eur J Cancer.

[33] Tateishi U, Gamez C, Dawood S, Yeung HW, Cristofanilli M, Macapinlac HA (2008). Bone metastases in patients with metastatic breast cancer: Morphologic and metabolic monitoring of response to systemic therapy with integrated PET/CT. Radiology.

